# A Novel Presentation of Metaphyseal Chondrodysplasia, Schmid Type with Factor VII Deficiency

**DOI:** 10.7759/cureus.7371

**Published:** 2020-03-23

**Authors:** Mushtaq Ahmed, Saad Nasir, Syeda Shaheera Riaz Hashmi, Zia Iqbal, Ayesha Saleem

**Affiliations:** 1 Pediatrics, Civil Hospital Karachi, Dow University of Health Sciences, Karachi, PAK; 2 Internal Medicine, United Medical and Dental College, Creek General Hospital, Karachi, PAK; 3 Internal Medicine, Civil Hospital Karachi, Dow University of Health Sciences, Karachi, PAK

**Keywords:** metaphyseal chondrodysplasia, factor-vii deficiency, skeletal deformities, short stature

## Abstract

Metaphyseal chondrodysplasia, Schmid type (MDSC) is a rare inherited autosomal disorder with characteristic skeletal deformities striking on radiological imaging, which includes metaphyseal cupping and fraying. Physical examination reveals short stature in early childhood, frontoparietal bossing, rachitic rosary, genu varum and valgum, and coxa vara usually. We believe that the constellation of clinical and radiographic findings of MDSC might look similar to vitamin D resistant rickets; hence, genetic analysis is needed to overcome diagnostic challenges faced by physicians to avoid unnecessary vitamin D supplementation in individuals. We report the first case of MDSC with a coexisting factor VII deficiency in an eight-year-old boy.

## Introduction

Metaphyseal chondrodysplasia, Schmid type (MDSC) is a rare autosomal dominant condition caused by a mutation in the COL10A1 gene [1]. It manifests in early childhood and results in a wide range of skeletal deformities. The affected child most commonly presents with short stature, coxa vara, genu varum and valgum, and a waddling gait. The incidence of MDSC is approximately three to six cases per 1,000,000 [2]. The diagnosis is made using radiological imaging, which reveals characteristic findings such as metaphyseal cupping and fraying, most evident in the lower extremities [3]. We present a case of an eight-year-old boy with MDSC and factor VII deficiency. Our literature review revealed that this is the first established case of co-occurrence of these two conditions in an individual. 

## Case presentation

An eight-year-old boy was brought by his father to the emergency department with a history of persistently active bleeding from his nose for an hour. The patient experienced several episodes of epistaxis, which occurred intermittently during the last 10 days. During these episodes, the bleeding stopped after pinching the nose for a few minutes. There was no history of bleeding from any other site. There was no parental consanguinity and family history of easy bruising, bleeding, or clotting disorder.

**Figure 1:**
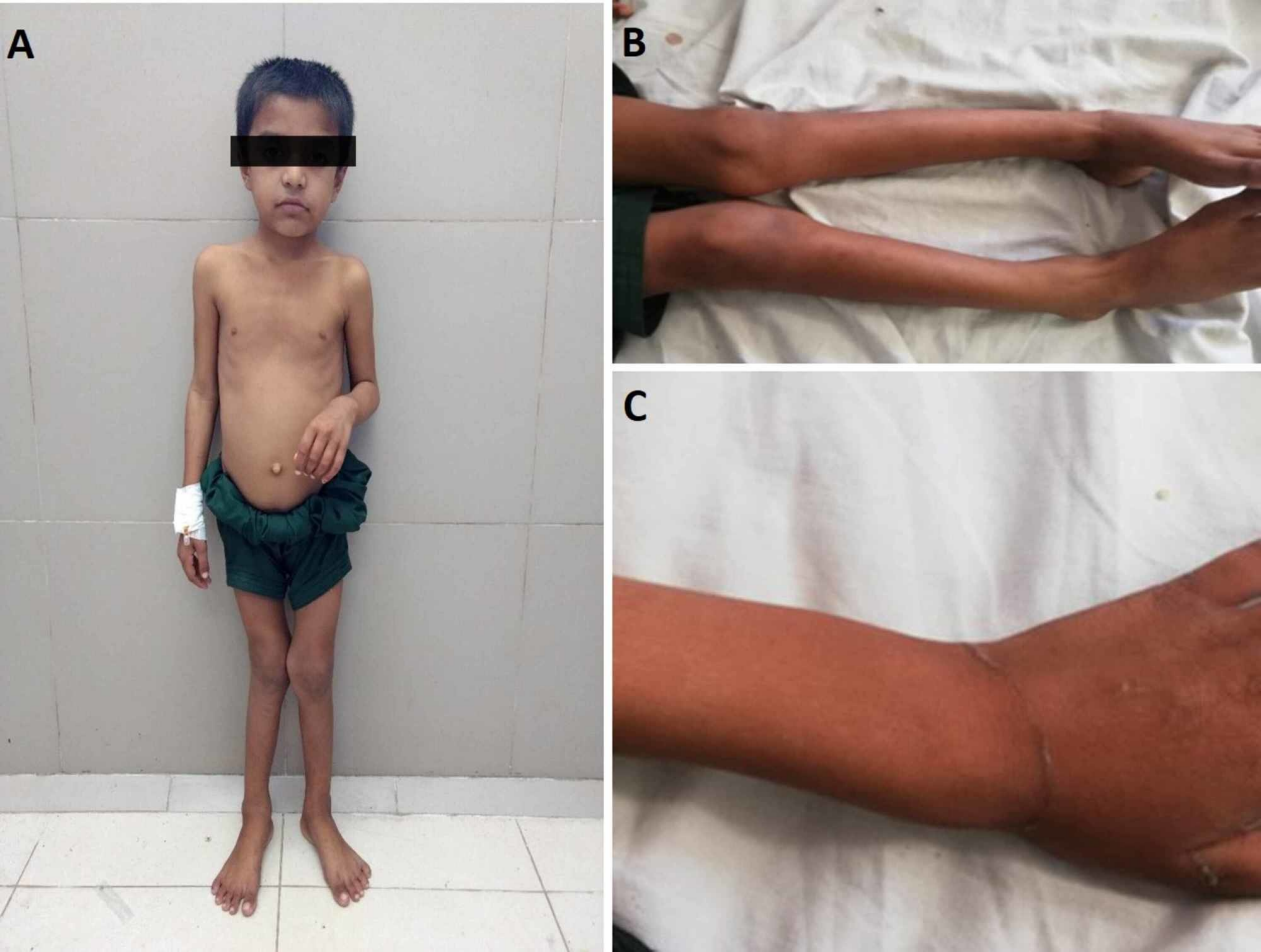
Photographs of the indexed patient at eight years of age (A) Frontoparietal head bossing. (B) Genu valgum (“knock-knees”) deformity. (C) Widened wrist.

The general physical exam showed that the child was pale, malnourished, and there was blood trickling from his nostrils. His vitals and blood pressure were within normal limits for his age and gender, and no overt clinical signs of heart failure noted. Pertinent examination findings included microcephaly (head circumference: 46 cm), short stature, frontoparietal head bossing (Figure 1A), rachitic rosary, genu valgum (Figure 1B), palpable widening of the wrists (Figure 1C), coxa vara, and a waddling gait. There was a presence of conjunctival pallor, indicating the presence of mild anemia.

Anthropometric measurements are presented in Table 1. 

**Table 1:** Anthropometric measurements of different body segments of the patient at eight years of age

Segments	Measurements	Percentile or standard deviation
Height	98 cm	Less than 3rd percentile
Weight	13.5 kg	Less than 3rd percentile
Head circumference	46 cm	Less than 3rd percentile
Arm span	100 cm	-
Upper segment	50 cm	-
Lower segment	40 cm	-
Upper segment: lower segment ratio	1.04	-

The patient was admitted to the pediatric unit for laboratory investigations and further management. Bleeding from nostrils was not entirely stopped after pressure was applied to the nose for about 15 minutes, and required placement of an epinephrine-soaked gauze as well. The ear, nose, and throat (ENT) on-call team was taken on-board for the likely need for packing but fortunately did not require any further interventions.

His routine laboratory findings were as follows: hemoglobin 4.8 mg/dL (normal range: 11-13 g/dL) and mean corpuscular volume 60 fL (normal range: 75-90 fL); total leukocyte count, blood urea nitrogen, creatinine, and electrolytes were in the normal reference range. His serum calcium 9.1 mg/dL (normal range: 9.4-10.3 mg/dL), phosphorus 2.1 mg/dL (normal range: 3.6-5.6 mg/dL), and alkaline phosphatase 756 IU/L (normal range: 44-147 IU/L) were deranged.

Nutritional profile was subsequently done and showed the following: decreased serum iron, 22 mcg/dL (normal range: 60-170 mcg/dL); decreased serum ferritin levels, 5 ng/mL (normal range: 12-300 ng/mL); increased total iron-binding capacity, 476 mcg/dL (normal range: 240-450 mcg/dL); normal serum folate, 6.8 ng/mL (normal range: 5-21 ng/mL); vitamin B12, 492 ng/mL (normal range: 200-900 ng/mL); and vitamin D levels 36 ng/mL. Clinical suspicion of vitamin D resistant rickets necessitated further biochemical workup, including urine phosphorus, urine calcium to creatinine ratio, and serum parathyroid hormone levels, which were also normal.

**Figure 2:**
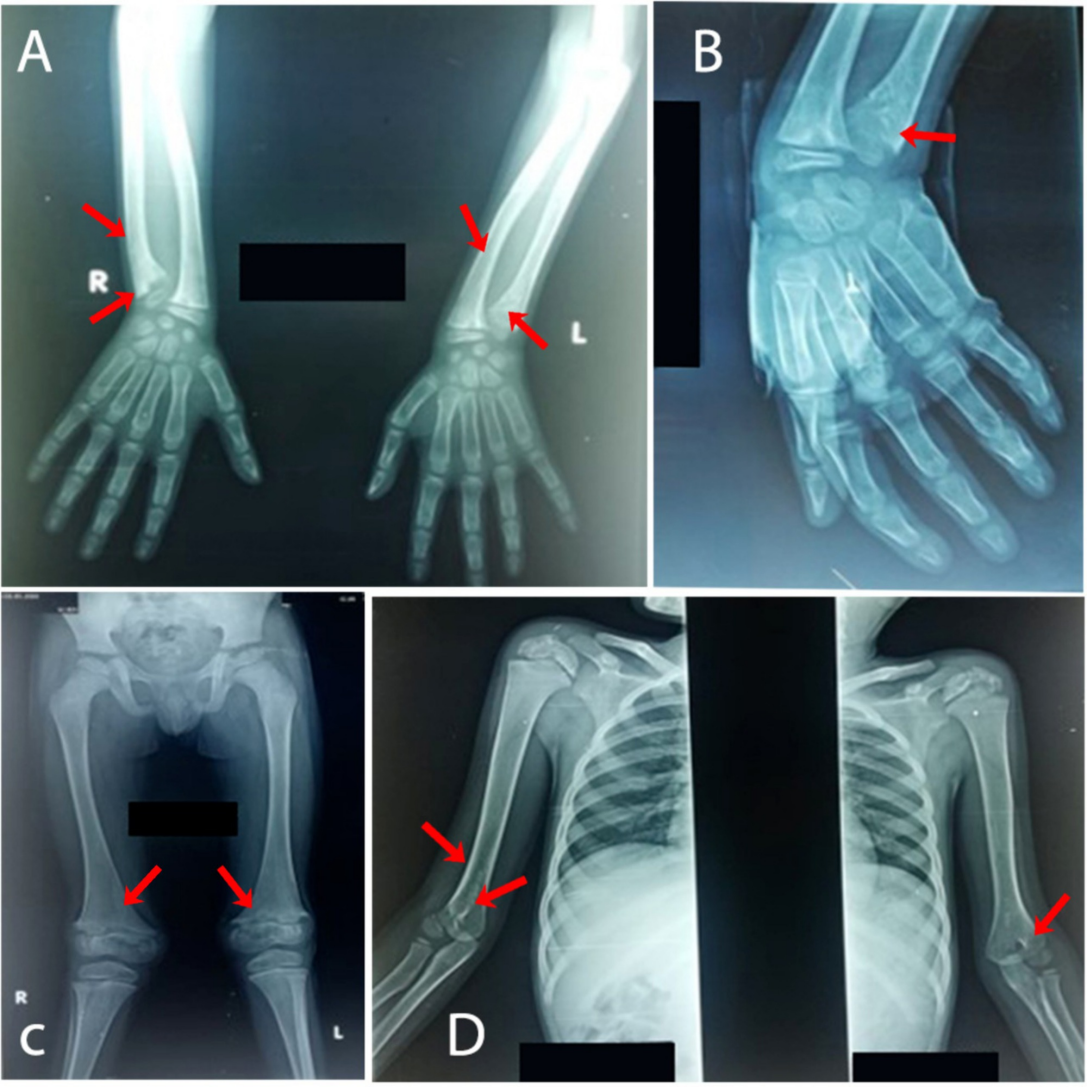
Conventional radiographs of the patient at eight years of age (A, B) X-ray of wrist joints showing irregularity and flaring at distal metaphysis of ulna bilaterally with bowing deformity; however, no fracture seen. (C) X-ray of pelvis and both femora showing irregularity at distal metaphysis of both femora without any fracture. (D) X-ray of both shoulders including humeri showing irregularity and flaring at proximal and distal humeral metaphysis with epiphyseal stippling.

Radiographical imaging showed significant bilateral symmetrical metaphyseal irregularities associated with deformities noted at shoulders, elbows, wrists, hips, knees, and ankle joints (Figure 2). Flaring of the metaphysis of bones of upper and lower limbs was also noted. There was a fragmentation of epiphysis in the shoulder and hip joints. Based on clinical and radiographical findings, MDSC was diagnosed. 

To further evaluate for a potential underlying bleeding disorder, coagulation studies were performed, which revealed a significantly prolonged prothrombin time of 70 seconds (normal range: 8.7-11.5 seconds) and international normalized ratio of 6 (normal: 1.1 or below), while activated partial thromboplastin time of 25 seconds was normal. Factor VII activity assay was subsequently done, which confirmed a coexisting congenital plasma factor VII deficiency, with plasma levels detected to be less than 6% (normal range: 65%-145%). The patient was managed with transfusion of recombinant factor VIIa, after which there was no subsequent bleeding episode noted. The parents were counseled about the condition and the associated factor VII deficiency. The patient was then referred for orthopedic consultation, and there was no immediate orthopedic concern. Furthermore, the patient was advised to frequent follow-ups to assess the likely need for supportive care in the future.

## Discussion

MDSC, described in 1949 by F. Schmid, is the most common and least serious variant of metaphyseal chondrodysplasias. This condition is associated with heterozygous mutations in the COL10A1 gene encoding for type X collagen with an autosomal dominant pattern of inheritance [4]. It is diagnosed with evidence of characteristic metaphyseal flaring and irregularity, which are most commonly found at the knees, coxa vara, and femoral bowing visible on radiographic imaging [5]. Although vitamin D resistant rickets and MDSC may be indistinguishable due to the similarity of clinical features, genetic analysis can be done to differentiate between the two conditions [6]. Dahl and Birkebaek reported a case of MDSC which was inappropriately treated for rickets because of the similarity of the two conditions [7].

Inherited factor VII deficiency is the most common of the rare inherited coagulation factor deficiencies and typically follows an autosomal recessive pattern of inheritance with an approximated prevalence of one case per 500,000 [8]. Mild cases of inherited factor VII deficiency present with epistaxis, while intracranial or gastrointestinal hemorrhage may occur in severe cases, which may be life-threatening. Coagulation profile, including factor VII activity, is used as a first-line diagnostic modality and can be followed up with factor VII immunoassay and genetic analysis [9].

To the best of our knowledge, this is the first report of MDSC associated with factor VII deficiency worldwide. There have been reports of MDSC from other regions, including India (three-year old girl), Venezuela (eight-year-old girl), and Japan (three-year-old boy) [1,6,10]. A cohort study done by Makitie et al. included 10 patients with confirmed MDSC, who were subsequently followed over time [11]. The study revealed that most patients presented with growth retardation during the second year of life. Short stature, waddling gait, and varus or valgus deformities were evident, which brought it to attention. Lower limbs were more significantly affected. The most common deformity was coxa vara, which was progressive and was managed by valgus osteotomy for surgical correction [11]. 

A case study done by Park et al., which included four patients with MDSC who had a male preponderance, revealed an equal number of genu valgum and varus deformity, while coxa vara and coxa magna were associated with three and one cases, respectively [12]. Management of MDSC usually involves a multidisciplinary approach with an orthopedic referral, physiotherapy, pain management, occupational therapy, and regular physical exercise to achieve healthy weight goals [13].

There has been one similar case study reported by Khorasani and Vakili in which MDSC presented with another inherited disorder, which was congenital adrenal hyperplasia; however, our case was different because there was a co-occurrence of factor VII deficiency, unlike any other reported cases in the literature [2].

## Conclusions

MDSC is a rare condition that manifests with short stature in early childhood. Physicians should be aware of the similarity of clinical features between MDSC and vitamin D resistant rickets to avoid unnecessary vitamin D supplementation. This case presented with a rare co-occurrence of factor VII deficiency with MDSC, which needs further studies to understand the possible correlation between two inherited disorders.
